# Phonological Underspecification: An Explanation for How a Rake Can Become Awake

**DOI:** 10.3389/fnhum.2021.585817

**Published:** 2021-02-17

**Authors:** Alycia E. Cummings, Ying C. Wu, Diane A. Ogiela

**Affiliations:** ^1^Department of Communication Sciences and Disorders, Idaho State University, Meridian, ID, United States; ^2^Swartz Center for Computational Neuroscience, University of California, San Diego, San Diego, CA, United States

**Keywords:** ERP, EEG, underspecification, MMN, ERSP, theta, gamma, phonology

## Abstract

Neural markers, such as the mismatch negativity (MMN), have been used to examine the phonological underspecification of English feature contrasts using the Featurally Underspecified Lexicon (FUL) model. However, neural indices have not been examined within the approximant phoneme class, even though there is evidence suggesting processing asymmetries between liquid (e.g., /ɹ/) and glide (e.g., /w/) phonemes. The goal of this study was to determine whether glide phonemes elicit electrophysiological asymmetries related to [consonantal] underspecification when contrasted with liquid phonemes in adult English speakers. Specifically, /ɹɑ/ is categorized as [+consonantal] while /wɑ/ is not specified [i.e., (–consonantal)]. Following the FUL framework, if /w/ is less specified than /ɹ/, the former phoneme should elicit a larger MMN response than the latter phoneme. Fifteen English-speaking adults were presented with two syllables, /ɹɑ/ and /wɑ/, in an event-related potential (ERP) oddball paradigm in which both syllables served as the standard and deviant stimulus in opposite stimulus sets. Three types of analyses were used: (1) traditional mean amplitude measurements; (2) cluster-based permutation analyses; and (3) event-related spectral perturbation (ERSP) analyses. The less specified /wɑ/ elicited a large MMN, while a much smaller MMN was elicited by the more specified /ɹɑ/. In the standard and deviant ERP waveforms, /wɑ/ elicited a significantly larger negative response than did /ɹɑ/. Theta activity elicited by /ɹɑ/ was significantly greater than that elicited by /wɑ/ in the 100–300 ms time window. Also, low gamma activation was significantly lower for /ɹɑ/ vs. /wɑ/ deviants over the left hemisphere, as compared to the right, in the 100–150 ms window. These outcomes suggest that the [consonantal] feature follows the underspecification predictions of FUL previously tested with the place of articulation and voicing features. Thus, this study provides new evidence for phonological underspecification. Moreover, as neural oscillation patterns have not previously been discussed in the underspecification literature, the ERSP analyses identified potential new indices of phonological underspecification.

## Introduction

Distinctive features are often described as the functional units of phonological systems (Chomsky and Halle, 1968). Phonemes are composed of combinations of features, with each phoneme being distinguished from all other phonemes by at least one feature. Phonological underspecification theories propose that only the distinctive features that differentiate a phoneme are present in the adult phonological representation (Kiparsky, 1985; Archangeli, 1988; Mohanan, 1991; Steriade, 1995). Specifically, underspecification identifies some features as “default” and others as “marked.” Default features are not stored within the phonological representation because they are assumed to be predictable by phonological rule. Conversely, marked features are the contrastive, or not otherwise predictable, phonological information that must be specified and stored. A marked phoneme is presumed to require the storage of more distinctive features in its phonological representation as compared to an unmarked phoneme. Thus, marked phonemes are considered to be more phonologically specified than unmarked phonemes.

By only storing specified features within the phonological representation, underspecification can improve speech processing efficiency when encountering the wide variability present in natural speech (Chomsky and Halle, 1968; Eulitz and Lahiri, 2004). Indeed, evidence for the effectiveness of phonological underspecification can be found in speech production. For example, phonological code retrieval in adults is slower when naming words beginning with marked phonemes, such as /ɹ/, as compared to unmarked phonemes, such as /b/ (Cummings et al., 2016).

The application of underspecification is also often observed in speech production errors. Specifically, speech errors typically affect specified features and phonemes rather than underspecified features and phonemes (Fromkin, 1973; Levelt et al., 1999; Brown, 2004). For example, approximants are involved in a common phonological process, called liquid gliding, found in the productions of both typically developing children and those with speech sound disorders (Shriberg, 1980; Broen et al., 1983). That is, many young English-speaking children incorrectly produce pre-vocalic /ɹ/ as [w] (e.g., ‘rake’ is pronounced as ‘wake’); however, children rarely, if ever, produce /w/ as [ɹ]^1^. Thus, during typical and atypical development, children tend to incorrectly produce phonemes with specified features (Stoel-Gammon and Dunn, 1985; Grunwell, 1987). Such evidence suggests that the underlying phonological representations can affect speech production. A better understanding of how specified and underspecified features are stored within phonological representations has important clinical implications for speech-language pathologists working with clients who have speech production errors. Due to the high frequency of the liquid gliding phonological process in pediatric American English-speaking populations, the examination of the /ɹ/-/w/ contrast is of particular interest.

As underlying phonological representations cannot be easily, if at all, accessed behaviorally, neuroimaging tools have proven useful in examining phonological underspecification. Neural markers of phonological underspecification have primarily been examined using the framework established by the Featurally Underspecified Lexicon (FUL) model (Lahiri and Marslen-Wilson, 1991; Lahiri and Reetz, 2002, 2010). Phonological underspecification has been found in vowels (Diesch and Luce, 1997; Eulitz and Lahiri, 2004; Cornell et al., 2011; Scharinger et al., 2012), as well as in consonants such as stops (Cummings et al., 2017), nasals (Cornell et al., 2013), and fricatives (Schluter et al., 2016). Many of these studies have indexed underspecification using the mismatch negativity (MMN), which is a well-studied event-related potential (ERP) peak that is elicited by auditory oddballs elicited within a stream of standard stimuli (Näätänen and Winkler, 1999; Picton et al., 2000; Näätänen et al., 2007). The MMN is a neurophysiological index of auditory change detection. As the deviant oddball becomes more different from the standard, MMN amplitude increases and latency decreases. Thus, the timing and size of the MMN may reflect the amount of perceived difference between the standard and the deviant stimuli (Tiitinen et al., 1994; Näätänen et al., 1997).

Within the FUL framework, the size of the MMN depends on the degree of specification of the features extracted from the stimuli (Winkler et al., 1999; Eulitz and Lahiri, 2004; Scharinger et al., 2011). For example, a true mismatch occurs when the more specified sound is the standard and the less specified sound is the deviant in the MMN oddball paradigm. In this situation, large MMN responses are elicited by the less specified deviant sound because it violates the feature expectations established by the standard. Conversely, a no-mismatch occurs when the less specified sound serves as the standard and the more specified sound is the deviant. In this context, no conflict between the phonetic features is identified because the feature was not specified by the standard. Thus, a very small, or no, MMN is elicited. Because of the predicted size differences of the MMN responses, the true mismatch contrast could be considered an easier feature comparison to make than the no-mismatch feature comparison.

Neural indices of underspecification have not been examined within the English approximant phoneme class, even though there is evidence suggesting processing asymmetries exist between liquid (e.g., /ɹ/) and glide (e.g., /w/) phonemes (Greenberg, 1975; Shriberg, 1980; Edwards, 1983; Clements, 1990). These asymmetries suggest that /ɹ/ and /w/ might differ in how they are stored within a phonological representation. While /ɹ/ and /w/ share several distinctive features (Chomsky and Halle, 1968), liquid phonemes are specified as [+consonantal] while glide phonemes are considered semi-vowels and are not specified for that feature (i.e., [–consonantal]. The basic definition of [consonantal] is: “… sounds [are] produced with a radical obstruction in the midsagittal region of the vocal tract; nonconsonantal sounds are produced without such an obstruction.” (Chomsky and Halle, 1968; p. 302). That is, [consonantal] phonemes are produced with varying amounts of constriction created by the labial, coronal, and/or dorsal articulators in the oral cavity. This feature classification essentially places vowels, glides, and laryngeal consonants in one natural sound class: [–consonantal], while the other consonant phonemes, including /ɹ/, are in a separate sound class: [+consonantal]. Thus, glide phonemes can be considered underspecified for [consonantal] in comparison to liquid phonemes.

While [consonantal] never functions as the sole feature responsible for distinguishing phonemes (Hume and Odden, 1996), it is hypothesized that constriction is the primary distinguishing feature of /ɹ/ and /w/, at least in American English. There are many ways that the American pre-vocalic /ɹ/ can be produced (Preston et al., 2020), with the /ɹ/ productions broadly described as either being “retroflex” or “bunched” in nature. Regardless of the type of production used, two separate constrictions are necessary for /ɹ/ to be produced: palatal constriction and pharyngeal constriction (Delattre and Freeman, 1968; Gick, 1999; Secord et al., 2007). The palatal constriction is made with the dorsum of the tongue being brought near the soft palate while the pharyngeal constriction is achieved with tongue root retraction. Indeed, problems with vocal tract constriction, and arguably the application of [consonantal], are often observed in children with speech sound disorders as they often have difficulty achieving adequate palatal and pharyngeal constriction necessary for an “accurate” /ɹ/ production. That is, they produce /ɹ/ with /w/-level constriction, which is not enough, either in terms of the amount and/or place of constriction.

Previous studies examining underspecification within the FUL-MMN paradigm relied on strict superset-subset relationships between the specified and underspecified features [e.g., contrasting voiced stops differing only by place of articulation: (coronal) vs. (labial); Cummings et al., 2017]. Such a contrast is not available for /ɹ/ and /w/ because they vary both in terms of manner and place of articulation. Thus, a strict application of FUL cannot be applied to identify the underspecification differences in /ɹ/ and /w/. Nevertheless, there are other potential ways to identify phonological underspecification in this contrast, which could then be tested using FUL-based predictions in an MMN paradigm.

Feature Geometry is an alternative way of organizing features in a hierarchical relationship that reflects the configuration of the vocal tract and articulators in a tree diagram. It allows for broad feature groupings (e.g., manner and place of articulation) to be associated with individual features. This means that some features, such as those at the root node (e.g., consonantal, sonorant) dominate place (e.g., coronal, labial) nodes^2^ (Bernhardt and Stoel-Gammon, 1994; Clements and Hume, 1995; Halle et al., 2000; Lahiri and Reetz, 2002). It is assumed that the determination of features in the higher nodes of the tree will impact the features available at lower nodes in the tree. Thus, the idea of markedness is present in both feature geometry and underspecification theory.

**Figure 1:**
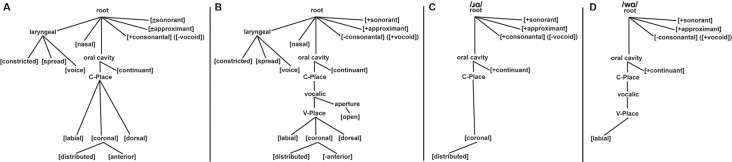
Feature geometry trees based on Clements and Hume (1995). Panel **(A)** displays the full feature geometry tree for consonants. Panel **(B)** displays the full feature geometry tree for vocoids (i.e., vowels and glides). Panel **(C)** displays the features of /ɹ/: [+consonantal] ([-vocoid]), [+sonorant], [+approximant], [+continuant], and [coronal: +distributed]. Panel **(D)** displays the features of /w/: [–consonantal] ([+vocoid]), [+sonorant], [+approximant], [+continuant], and [labial].

Given the hypothesis that constriction is the distinguishing articulatory property of /w/ and /ɹ/, the feature geometry theory of Clements and Hume (1995) was used (Figure 1). The Clements and Hume (1995) model is a constriction-based approach that defines most phonemes in terms of their constriction location and degree. This means that the place features (i.e., the articulators and dependents) define the constriction location while the articulator-free features define constriction degree (i.e., consonantal/vocoid, sonorant, approximant, and continuant). Three major class features are located at the root node: [sonorant], [approximant], and [vocoid]. As [vocoid] is the terminal opposite of [consonantal], we will refer to /w/ as [–consonantal] ([+vocoid]) and /ɹ/ as [+consonantal] ([-vocoid]). These distinct [+consonantal] and [–consonantal] designations place /ɹ/ on the C-place tier and /w/ on the V-place tier, respectively. Both phonemes are [+sonorant], [+approximant], and [+continuant].

In this model, the place nodes for vowels and consonants are on separate tiers, designated *V*-place and *C*-place, respectively, with the vocalic node linking under the *C*-place node. The actual constriction location (i.e., place of articulation) is largely the same for both vowels and consonants: [labial], [coronal], and [dorsal]. As a result, consonant and vowel articulators are placed on the same tier. In addition, [coronal] has two dependents: [anterior] and [distributed]. This means that coronal itself is not the terminal place of articulation—[anterior] or [distributed] is; conversely, [labial] and [dorsal] are terminal. This feature tree organization leads to /w/ being characterized as [–consonantal, labial] while /ɹ/ contains the features [+consonantal, coronal: +distributed]. With [coronal: +distributed] being located lower on the feature tree than [labial], /ɹ/ more specified for the place of articulation than /w/. Thus, following Clements and Hume (1995), as compared to /w/, /ɹ/ is more specified both in terms of the manner of articulation [+consonantal] and place of articulation [coronal: +distributed].

In regards to /ɹ/ and /w/, the feature [consonantal] ([vocoid]) is located on the highest node of the Clements and Hume (1995) tree (Figure 1). As such, processing this feature should dominate the processing of features at lower nodes, including the place of articulation nodes (i.e., *C*-Place and *V*-Place). That is, feature geometry theory predicts that the presence or absence of the [consonantal] feature will be the relevant contrasting feature of /w/ and /ɹ/. While the Clements and Hume (1995) model does not have the same organization as FUL, both can identify features and phonemes that are less specified than others. It is assumed that if underspecification is a language universal phenomenon, the underspecification-specific MMN predictions of FUL would hold regardless of whether FUL was strictly adhered to, or if another theoretical interpretation of underspecification was used. Thus, the Clements and Hume (1995) model was employed as the framework for the [consonantal] underspecification of /w/, as compared to /ɹ/. This prediction was then tested in the present study using the FUL-based predictions in an MMN paradigm.

Feature geometry can also provide a framework to explain children’s acquisition of phonemes and speech production errors. That is, higher and dominant nodes in the hierarchy are proposed to be acquired before subordinate nodes (Bernhardt, 1992; Core, 1997). Moreover, default features would be acquired early in development, with minimal specification present in the phonological representations (Bernhardt and Gilbert, 1992). The liquid gliding phonological process could then be explained by the early acquisition of /w/, which is not specified for [consonantal] and is the default feature. Only after the [consonantal] feature of /ɹ/ is fully established in the phonological representation is the gliding pattern suppressed in children’s production. As the basic definition of [consonantal] suggests that articulatory precision (i.e., constriction control) is necessary, it seems logical that an underspecified [–consonantal] phoneme (i.e., /w/) would be acquired prior to a [+consonantal] phoneme (i.e., /ɹ/). Thus, there is speech production evidence for the underspecification of [consonantal] in typical and atypical development.

There is clear evidence from developmental and clinical (i.e., disordered speech) data that there is a relationship between /w/ and /ɹ/ in American English, with young children and children with speech disorders substituting [w] for /ɹ/. Moreover, when adults mimic the speech of young children, they almost always substitute [w] for /ɹ/. Thus, the liquid gliding phonological process is an arguably ingrained stereotype of young children’s speech—even for adults who have essentially no explicit knowledge of the phonological system. Given these observations, it was hypothesized that /w/ contains one or more default features leading to its common usage in development while /ɹ/ contains one or more specified features that limits its production early in development. The purpose of the study was to address this potential underspecified/specified feature relationship in adults before examining the neural processing patterns in children. Thus, this study aims to determine whether glide phonemes elicit [consonantal] underspecification-related electrophysiological asymmetries when contrasted with liquid phonemes in adult English speakers.

Following FUL’s predictions and framework, if /w/ is less specified than /ɹ/ in terms of the manner of articulation, the former phoneme should elicit a larger MMN response than the latter phoneme. That is, a standard stream of /w/ phonemes would not set expectations for [consonantal], so when a deviant /ɹ/ is presented, it would be a no-mismatch. Thus, a small, or no, MMN response is predicted to occur in the no-mismatch situation. Conversely, hearing /ɹ/ as the standard stimulus would set up the expectation for [+consonantal], which would be violated by a deviant /w/. Thus, a large MMN response is predicted to occur in the true mismatch situation.

While underspecification has primarily been addressed with ERPs, subtle processing differences between distinct phonemes may not be detected due to the averaging of brain signals in traditional ERP methods. In contrast, time-frequency analyses provide an alternative approach that involves decomposing the spectral power of the EEG signal over time (Davidson and Indefrey, 2007; Cohen, 2014). Unlike ERPs, which only reveal phase-locked changes in the time series data, this approach affords both a view of changes in EEG signals that are phase-locked to stimulus onset (evoked responses), as well as a view of changes that are not phase-locked (induced responses). The synchronization of neuronal cell assemblies proposed to underlie increases in induced power has been hypothesized to mediate the binding of perceptual information (Singer and Gray, 1995). Experimental results have also implicated induced responses in various cognitive functions such as working memory (Gurariy et al., 2016) and attentional processes (Ward, 2003).

In keeping with these findings, there is reason to believe that phonological underspecification could also be indicated by neural oscillation patterns. For example, cortical oscillations in the theta (~4–7 Hz) and low gamma (~25–35 Hz) bands have been implicated in decoding syllabic and phonemic segments, respectively, from continuous speech (Luo and Poeppel, 2007; Ghitza, 2011; Giraud and Poeppel, 2012; Doelling et al., 2014; Di Liberto et al., 2015). That is, theta band has been proposed to represent higher-order syllable-level processing while low gamma band activities have been linked to phoneme feature-level processing (e.g., formant transitions, voicing). Possibly, one, or both, of these bands could demonstrate underspecification response asymmetries. As neural oscillation patterns underlying phonological underspecification have not previously been examined, this work was exploratory in nature and no specific hypotheses were proposed regarding theta and low gamma response patterns.

## Materials and Methods

### Participants

Fifteen native speakers of (American) English (three males, 12 female; mean age: 21.71 years, range: 19–26 years) who were undergraduate students participated in the study. All of them had a normal or corrected-to-normal vision, and none had a history of speech, language, and/or hearing impairment. This study was approved by the university institutional review board and each participant signed informed consent following the university human research protection program.

### Stimuli

Syllables (consonant + /ɑ/) were pronounced by a male North American English speaker. The syllables were digitally recorded in a sound isolated room (Industrial Acoustics Company, Inc., Winchester, UK) using a Beyer Dynamic (Heilbronn, Germany) Soundstar MK II unidirectional dynamic microphone and Behringer (Willich, Germany) Eurorack MX602A mixer. All syllables were digitized with a 16-bit AD converter at a 44.1 kHz sampling rate. The average intensity of all the syllable stimuli was normalized to 65 dB SPL.

The adults heard two oddball stimulus sets, each containing the same four English speech consonant-vowel (CV) syllables: “ra” (/ɹɑ/), “wa” (/wɑ/), “ba” (/bɑ/), and “da” (/dɑ/). In one stimulus set, /ɹɑ/ served as the standard syllable, with the other three CV syllables serving as deviants. In the second stimulus set, /wɑ/ served as the standard syllable, with the other three syllables being deviants. Only responses to the /ɹɑ/ and /wɑ/ syllables will be addressed further since they served as both standard and deviant stimuli, which allowed for the creation of same-stimulus identity difference waves. Since /bɑ/ and /dɑ/ deviants were incorporated to prevent MMN habituation, they were not examined. As initially recorded, the syllables varied slightly in duration, due to the individual phonetic make-up of each consonant. Syllable duration was minimally modified in /wɑ/ (by shortening the steady-state vowel duration by 24 ms) so that all syllables were 375 ms in length. Each syllable token used in the study was correctly identified by at least 15 adult listeners.

The phonotactic probability^3^ of each phoneme and syllable were calculated using the online phonotactic probability calculator^4^ (Vitevitch and Luce, 2004). These probability values are presented in Table 1. The singleton /ɹ/ occurs 2.5 times more frequently than /w/ in English. Similarly, the ɹɑ/ syllable in English occurs 1.375 times more frequently than that of /wɑ/.

**Table 1:** The phonotactic probability in English of the phonemes and syllables used in the study.

	Consonant	Consonant + /ɑ/
/ɹ/	0.0501	0.0011
/w/	0.0203	0.0008
/b/	0.0512	0.0039
/d/	0.0518	0.0023

### Stimulus Presentation

The stimuli were presented in blocks containing 237 standard stimuli and 63 deviant stimuli (21 per deviant), with five blocks of each stimulus set being presented to each participant (10 total blocks). The stimulus sets were presented sequentially in the session, with all five blocks of one stimulus set (e.g., /ɹɑ/ standard set) being presented before the other stimulus set (e.g., /wɑ/ standard set); the presentation of the stimulus sets was counterbalanced across participants. Each block lasted approximately 6 min and the participants were given a break between blocks when necessary. Within the block, the four stimuli were presented using an oddball paradigm in which the three deviant stimuli (probability = 0.07 for each) were presented in a series of standard stimuli (probability = 0.79). Stimuli were presented in a pseudorandom sequence and the onset-to-onset inter-stimulus interval varied randomly between 600 and 800 ms. The syllables were delivered by stimulus presentation software (Presentation software, www.neurobs.com). The syllable sounds were played *via* two loudspeakers situated 30 degrees to the right and left from the midline 120 cm in front of a participant, which allowed the sounds to be perceived as emanating from the midline space. The participants sat in a sound-treated room and watched a silent cartoon video of their choice. The recording of the ERPs took approximately 1 h.

### EEG Recording and Averaging

Sixty-six channels of continuous EEG (DC-128 Hz) were recorded using an ActiveTwo data acquisition system (Biosemi, Inc, Amsterdam, Netherlands) at a sampling rate of 256 Hz. This system provides “active” EEG amplification at the scalp that substantially minimizes movement artifacts. The amplifier gain on this system is fixed, allowing ample input range (−264 to 264 mV) on a wide dynamic range (110 dB) Delta- Sigma (ΔΣ) 24-bit AD converter. Sixty-four channel scalp data were recorded using electrodes mounted in a stretchy cap according to the International 10-20 system. Two additional electrodes were placed on the right and left mastoids. Eye movements were monitored using FP1/FP2 (blinks) and F7/F8 channels (lateral movements, saccades). During data acquisition, all channels were referenced to the system’s internal loop (CMS/DRL sensors located in the centro-parietal region), which drives the average potential of a subject (the Common Mode voltage) as close as possible to the Analog-Digital Converter reference voltage (the amplifier “zero”). The DC offsets were kept below 25 microvolts at all channels. Off-line, data were re-referenced to the common average of the 64 scalp electrode tracings.

Data processing followed an EEGLAB (Delorme and Makeig, 2004) pipeline. Briefly, data were high-pass filtered at 0.5 Hz using a pass-band filter. Line noise was removed using the CleanLine EEGLAB plugin. Bad channels were rejected using the trimOutlier EEGLAB plugin and the removed channels were interpolated. Source level contributions to channel EEG were decomposed using Adaptive Mixed Model Independent Component Analysis (AMICA; Palmer et al., 2008) in EEGLAB^5^. Artifactual independent components (ICs) were identified by their activation patterns, scalp topographies, and power spectra, and the contribution of these components to the channel EEG was zeroed (Jung et al., 2000; Delorme and Makeig, 2004). Epochs containing 100 ms pre-auditory stimulus to 800 ms post-auditory stimulus time were baseline-corrected for the pre-stimulus interval and averaged by stimulus type. On average, individual data contained 804 (SD = 84) /ɹɑ/ standard syllable epochs (i.e., trials), 794 (SD = 79) /wɑ/ standard syllable epochs, 96 (SD = 9) /ɹɑ/ deviant syllable epochs, and 97 (SD = 9) /wɑ/ deviant syllable epochs.

### ERP and EEG Measurements

Three different data analysis strategies were used in the present study: (1) traditional mean amplitude repeated measure ANOVA analyses using averaged data; (2) cluster-based permutation analyses of averaged data (Bullmore et al., 1999; Groppe et al., 2011); and (3) event-related spectral perturbation (ERSP) analyses (Makeig, 1993).

#### Mean Amplitude Measurements of Averaged Data

The dual stimulus set nature of the present study allowed for the creation of “same-stimulus”, or identity, difference waveforms. These difference waves were created by subtracting the ERP response of a stimulus serving as the standard from that of the same stimulus serving as the deviant, across stimulus sets. For example, the ERP response for /ɹɑ/ as the standard was subtracted from the ERP response for /ɹɑ/ as the deviant (of the reversed stimulus set; Eulitz and Lahiri, 2004; Cornell et al., 2011, 2013). The creation of identity difference waveforms eliminates the potential confound that may result from acoustic stimulus differences since the same stimulus is used to elicit both the standard and deviant responses. The waveforms were visually inspected from 0 to 400 ms, with the MMN appearing between approximately 100 and 250 ms post-syllable onset.

Since the MMN was present in 12 electrodes centered around the scalp midline (Fz, F1/F2, FCz, FC1/FC2, Cz, C1/C2, CPz, and CP1/CP2)^6^, these electrodes were selected for the mean amplitude analyses. The MMN elicited by /wɑ/ extended for approximately 150 ms. Given the extended duration of the MMN, the mean amplitude measurement of the MMN was split into three 50 ms windows: 100–150, 150–200, and 200–250 ms post-stimulus onset. Phonological underspecification in the identity difference waves was analyzed separately in each time window using a Phoneme Type (/ɹɑ/, /wɑ/) × Anterior-Posterior (4 Levels) × Left-Right (3 Levels) repeated measure ANOVA.

Since the difference waves were generated from the standard and deviant syllable ERPs, the mean amplitude measurements of the standard and deviant waveforms were taken from the same three time windows as that of the MMN: 100–150, 150–200, and 200–250 ms post-syllable onset. In terms of ERP waveform morphology, these measurements approximately captured the auditory N1 (100–200 ms) and auditory P2 (200–250 ms). Phonological underspecification in these ERPs was analyzed separately for each time window using a Phoneme Type (/ɹɑ/, /wɑ/) × Trial Type (Standard, Deviant) × Anterior-Posterior (4 Levels) × Left-Right (3 Levels) repeated measure ANOVA. Partial eta squared (*η*^2^) effect sizes are reported for all significant effects and interactions. When applicable, Geiser–Greenhouse corrected *p*-values are reported.

#### Cluster Mass Permutation Tests of Averaged Data

The ERPs were submitted to repeated measures two-tailed cluster-based permutation tests (Bullmore et al., 1999; Groppe et al., 2011) using the Mass Univariate ERP Toolbox for EEGLAB^7^. Four tests were conducted: (1) /ɹɑ/ standard vs. /ɹɑ/ deviant ERPs; (2) /wɑ/ standard vs. /wɑ/ deviant ERPs; (3) /ɹɑ/ vs. /wɑ/ standard ERPs; and (4) /ɹɑ/ vs. /wɑ/ deviant ERPs. Each test included the same 12 electrodes from the mean amplitude ERP measurements: F1/F2, Fz, FC1/FC2, FCz, C1/C2, Cz, CP1/CP2, and CPz. All of the time points (measured every 4 ms; 155 total time points) between 0 and 600 ms at the 12 scalp electrodes were included in the test (i.e., 1,860 total comparisons).

*T*-tests were performed for each comparison using the original data and 2,500 random within-participant permutations of the data. For each permutation, all *t*-scores corresponding to uncorrected *p*-values of 0.05 or less were formed into clusters. Electrodes within about 5.44 cm of one another were considered spatial neighbors, and adjacent time points were considered temporal neighbors. The sum of the *t*-scores in each cluster was the “mass” of that cluster. The most extreme cluster mass in each of the 2,501 sets of tests was recorded and used to estimate the distribution of the null hypothesis (i.e., no difference between conditions). The permutation cluster mass percentile ranking of each cluster from the observed data was used to derive *p*-values assigned to each member of the cluster. *t*-scores that were not included in a cluster were given a *p*-value of 1.

#### Event-Related Spectral Perturbation (ERSP) Analyses

ERSP analyses were performed to examine theta (4–7 Hz) and low gamma (25–35 Hz) band activities elicited by the /ɹɑ/ and /wɑ/ standard and deviant syllable stimuli. This approach was informed by prior work on speech syllable decoding (Ghitza, 2011; Giraud and Poeppel, 2012). ERSPs were computed from time-series data from 16 electrodes: F3/F4, F1/F2, FC3/FC4, FC1/FC2, C3/C4, C1/C2, CP3/CP4, CP1/CP2^8^ (Supplementary Figure 1). Data were epoched from −0.6 ms before stimulus onset to 1.6 ms after. Estimates of spectral power for each of these EEG epochs were computed across 200 equally spaced time points along 100 frequency steps spanning 3–50 Hz using Morlet wavelets with cycles gradually increasing with frequency (Delorme and Makeig, 2004). ERSPs were created by converting spectral density estimates to log power, averaging across single trials, and subtracting the mean log power derived from the pre-stimulus baseline period of the same trials. The final output for each channel was a matrix of 100 frequency values (3–50 Hz) by 200 time points (−0.5 to 1 s).

It has been proposed that the decoding of auditory information during speech perception occurs during two distinct time scales—one which relates to syllable-level processing (~200 ms) and one related to phoneme-level processing (~25 ms; Poeppel, 2003; Ghitza, 2011; Giraud and Poeppel, 2012; Doelling et al., 2014). As such, theta (4–7 Hz) bandwidth responses were measured in one 200 ms window occurring 100–300 ms post-syllable onset. Low gamma (25–35 Hz) bandwidth responses were measured separately in five 50 ms windows occurring 50–300 ms post-syllable onset.

For each participant, the magnitude of synchronized theta and gamma activity at each electrode was derived by averaging estimates of spectral power computed across steps within each of these bandwidths and across time points within the selected time interval. Phoneme-related differences in theta and low gamma power were examined in separate Phoneme Type (/ɹɑ/, /wɑ/) × Trial Type (Standard, Deviant) × Laterality (Left, Right) × Anterior-Posterior (4) × Electrode Laterality (Far, Close) repeated measure ANOVAs.

## Results

Only significant results for all analyses are reported.

### ERP Mean Amplitude Results

**Figure 2:**
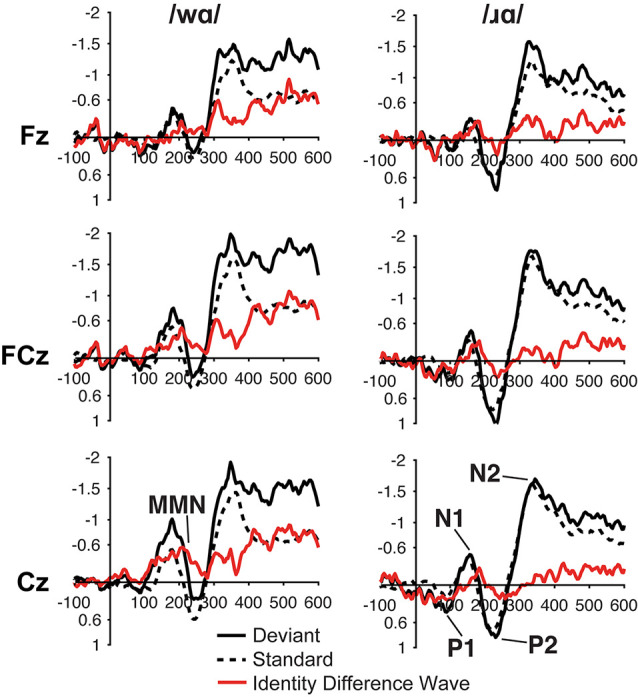
Event-related potential (ERP) waveforms elicited by the /wɑ/ (left side) and /ɹɑ/ (right side) syllables in the ERP study. The deviant waveforms represent the neural responses when the deviant syllable was presented within a stream of the opposite syllable standards. Subtracting the standard syllable response from the deviant syllable response resulted in the identity difference waves. Note that negative is plotted *up* in all waveforms.

For both the /ɹɑ/ and /wɑ/ syllables, the ERP waveforms elicited by the standard and deviant stimuli consisted of P1 at ca. 75 ms, N1 at ca. 150 ms, P2 at ca. 225 ms, and N2 at ca. 350 ms (Figure 2). In the same-stimulus identity difference waves, an MMN was visible in both the /ɹɑ/ and /wɑ/ identity waveforms at ca. 200 ms; the /wɑ/ MMN extended from ca. 100–250 while the /ɹɑ/ MMN extended from ca. 175–225 ms (Figure 2).

#### Identity Difference Waves: MMN

**Figure 3:**
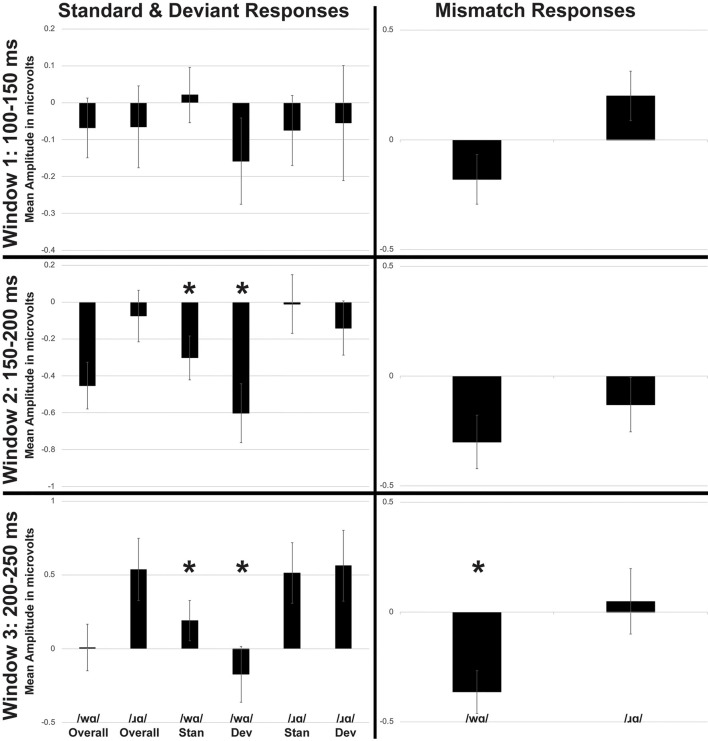
Average mean amplitudes for standard and deviant ERPs (left side) and mismatch responses measured in identity difference waves (right side) across three time windows: (1) 100–150 ms post-syllable onset; (2) 150–200 ms post-syllable onset; and (3) 200–250 ms post-syllable onset. Error bars represent SEM. Time Windows 1 and 2 broadly captured the auditory N1 response, while Time Window 3 captured the auditory P2 response. The Mismatch Negativity (MMN) was present in all three time windows. The /wɑ/ deviants were significantly more negative than the /wɑ/ standards during Window 3. The MMN responses elicited by /wɑ/ were significantly more negative than those elicited by /ɹɑ/ during Window 3. *Significant effects.

Individual participants’ mean ERP responses to /ɹɑ/ and /wɑ/ stimuli are presented in Supplementary Figure 2. During the 200–250 time window, MMN responses elicited by /wɑ/ were significantly more negative than those elicited by /ɹɑ/ (*F*_(1,14)_ = 5.479, *p* < 0.04, *η*^2^ = 0.281; Figures 2, 3). During the 150–200 ms time window, the overall magnitude of the MMN was larger over the left hemisphere, as compared to the right (*F*_(2, 28)_ = 5.343, *p* < 0.02, *η*^2^ = 0.276; Supplementary Figure 3).

#### Standard and Deviant Waveforms

The standard and deviant ERP responses elicited by /wɑ/ were significantly more negative than those elicited by /ɹɑ/ during both the 150–200 ms time window (*F*_(1,14)_ = 12.448, *p* < 0.004, *η*^2^ = 0.471) and 200–250 ms time window (*F*_(1,14)_ = 21.272, *p* < 0.0001, *η*^2^ = 0.603; Figure 3 and Supplementary Figure 4). Deviant trials elicited significantly more negative responses than did standard responses during the 150–200 ms time window (*F*_(1,14)_ = 10.029, *p* < 0.008, *η*^2^ = 0.417).

A phoneme × trial type interaction (*F*_(1,14)_ = 5.481, *p* < 0.04, *η*^2^ = 0.281) was observed during the 200–250 ms time window (Figure 3). Whereas the /ɹɑ/ standard and /ɹɑ/ deviant responses did not reliably differ, ERPs elicited by /wɑ/ deviants were consistently more negative than the /wɑ/ standards (*F*_(1,14)_ = 14.189, *p* < 0.003, *η*^2^ = 0.503).

#### ERP Summary

The FUL underspecification paradigm predicts that the underspecified phoneme deviant presented within a stream of the specified phoneme standards will elicit a large MMN response, as this situation creates a true feature mismatch context. The opposite stimulus presentation is predicted to elicit a small, or no, MMN response due to the feature no-match context. These hypotheses were supported. The underspecified /wɑ/ stimuli elicited significantly larger and more negative responses than did the specified /ɹɑ/.

### Cluster Permutation Analysis Results

**Figure 4:**
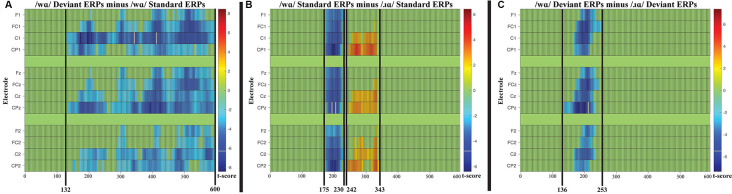
**(A)** Raster diagram illustrating differences between the /wɑ/ deviants and standards, which extended from 132 ms post-syllable onset to the end of the analysis window (600 ms). **(B)** Raster diagram illustrating differences between /wɑ/ standards and /ɹɑ/ standards, one cluster extended from 175 to 230 ms post-syllable onset and the second cluster extended from 243 to 343 ms post-syllable onset. **(C)** Raster diagram illustrating differences between /wɑ/ deviants and /ɹɑ/ deviants, which extended from 136 to 253 ms post-syllable onset. There were no reliable clusters for the comparison of /ɹɑ/ deviants and standards. Note: for the raster diagrams, colored rectangles indicate electrodes/time points in which the ERPs to one stimulus are significantly different from those to another. The color scale dictates the size of the *t*-test result, with dark red and blue colors being more significant. Green areas indicate electrodes/time points at which no significant differences were found. Note that the electrodes are organized along the *y*-axis somewhat topographically. Electrodes on the left and right sides of the head are grouped on the figure’s top and bottom, respectively; midline electrodes are shown in the middle. Within those three groupings, the *y*-axis top-to-bottom corresponds to scalp anterior-to-posterior.

Four cluster-level mass permutation tests encompassing 0–600 ms were applied to the standard and deviant syllable data. The results of the tests are displayed in raster diagrams in Figures 4A–C.

No reliable clusters were identified when examining the difference between /ɹɑ/ standards and /ɹɑ/ deviants. On the other hand, one broadly distributed cluster extending from 132 to 600 ms signified a period during which the /wɑ/ deviants elicited more negative ERP responses than the /wɑ/ standards; the smallest significant *t*-score (in absolute values) was: *t*_(14)_ = −2.159, *p* < 0.0001 (Figure 4A).

When contrasting the standard syllables, two broadly distributed clusters extending from 175 to 230 ms and 242 to 343 ms signified two time periods during which the /ɹɑ/ standards differed from the /wɑ/ standards (Figure 4B); the smallest significant *t*-score was: *t*_(14)_ = 2.149, *p* < 0.05. When the /ɹɑ/ and /wɑ/ deviant syllables were contrasted, a broadly distributed cluster extending from 136 to 253 ms signified a time period during which the /wɑ/ deviants elicited more negative (i.e., larger) ERP responses than the /ɹɑ/ deviants (Figure 4C); the smallest significant *t*-score was: *t*_(14)_ = 2.158, *p* < 0.005.

#### Cluster Permutation Analysis Summary

FUL predicts a larger MMN will be elicited by an underspecified phoneme, as compared to a specified phoneme. Consistent with the ERP analyses, this prediction was confirmed. The MMN appeared in the difference waveforms between 100 and 300 ms post-syllable onset. The effects seen in the /wɑ/ stimuli extended far beyond the traditional timeline of the MMN^9^. This result was unexpected. As no phoneme type differences were observed in the standard trial and deviant trial analyses, this effect appears to be specific to the contrast of the /wɑ/ standard and deviant trials. Visual analysis of the cluster permutation (Figure 4A) suggests that there were potentially three parts to the /wɑ/ effect, ~132–~275, ~300–400, and ~400–600 ms. Thus, the first part could be attributed to the MMN, the second part could represent the deviance-related or novelty N2 (Folstein and Van Petten, 2008), while the third part could be attributed to a late MMN or Late Negativity (LN)^10^. Previous studies have identified the LN as a secondary index of speech perception and discrimination (Korpilahti et al., 1995; Čeponienė et al., 1998; Cheour et al., 1998; Shafer et al., 2005; Datta et al., 2010; Hestvik and Durvasula, 2016). However, there is currently insufficient information in the underspecification literature to further interpret this finding.

Consistent with the ERP analyses, phoneme type differences in the standard and deviant trials were observed. The two clusters in the analysis of standard trials were consistent with the auditory N1 and P2 ERP responses. That is, in the first period, the /wɑ/ standards elicited a larger auditory N1 than did the /ɹɑ/ standards. During the second period, the /ɹɑ/ standards elicited a larger auditory P2 than did the /wɑ/ standards. Similarly, in the analysis of deviant trials, the identified cluster almost exclusively encompassed the auditory N1 ERP response. As the MMN is derived from the subtraction of the standard stimulus from the deviant stimulus, the responses elicited by /wɑ/ are consistent with the prediction that the underspecified phoneme should elicit larger (i.e., more negative) responses than the more specified /ɹɑ/. Thus, the cluster permutation analyses provide converging evidence for the underspecification of /wɑ/.

### ERSP Results

#### Theta Band (4–7 Hz) 100–300 ms

**Figure 5:**
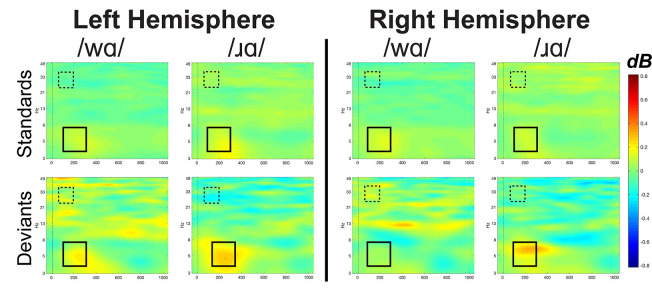
Event-related spectral perturbation (ERSP) activation patterns (in dB) elicited by /wɑ/ and /ɹɑ/ in the standard and deviant stimuli averaged across the eight left hemisphere electrodes and eight right hemisphere electrodes for theta (4–7 Hz) and low gamma (25–35 Hz) bandwidths. Time is on the *x*-axis and frequency is on the *y*-axis. Theta band window of interest is highlighted by the solid black box while the low gamma band window of interest is highlighted by the dashed box. Overall, /ɹɑ/ elicited greater neural synchrony (i.e., more activation) in the theta band than did /wɑ/. The /ɹɑ/ deviant elicited less neural synchrony over the left hemisphere, as compared to the right, in the low gamma band.

**Figure 6:**
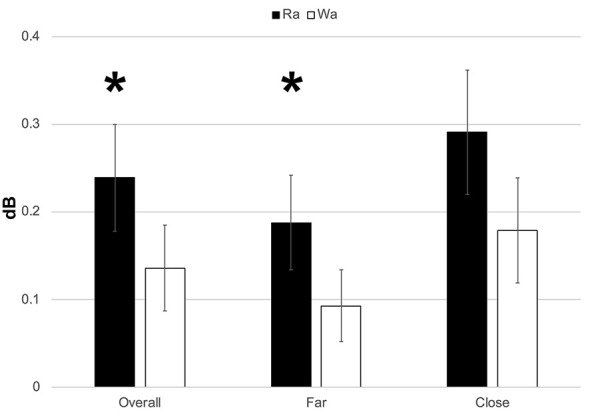
ERSP activation (in dB) elicited by /wɑ/ and /ɹɑ/ for the theta (4–7 Hz) bandwidth in the 100–300 ms time window. The /ɹɑ/ elicited greater neural synchrony (i.e., more activation) in the theta band than did /wɑ/. The electrodes closer to midline (e.g., F1 and F2) elicited greater theta activation than did the electrodes further away from midline (e.g., F3 and F4). *Significant effects.

Individual participants’ mean theta band responses to /ɹɑ/ and /wɑ/ standards and deviants are presented in Supplementary Figure 5. Theta responses elicited by /ɹɑ/ were significantly greater than those elicited by /wɑ/ (*F*_(1,14)_ = 4.571, *p* = 0.05, *η*^2^ = 0.246; Figures 5, 6). A significant electrode laterality effect was found (*F*_(1,14)_ = 14.053, *p* < 0.003, *η*^2^ = 0.501), as the electrodes closer to midline (1- and 2-level electrodes; *M* = 0.235, SEM = 0.059) elicited greater theta activity than did the far lateral electrodes (three- and four-level electrodes; *M* = 0.140, SEM = 0.043).

#### Low Gamma Band (25–35 Hz) 50–300 ms

**Figure 7:**
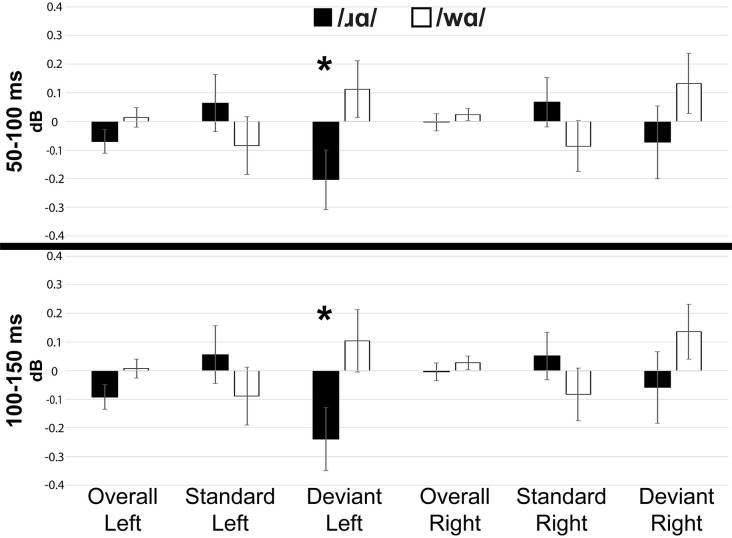
Event-related spectral perturbation activation (in dB) elicited by /wɑ/ and /ɹɑ/ for the low gamma (25–35 Hz) bandwidth across five 50 ms time windows: 50–100 ms, 100–150 ms, 150–200 ms, 200–250 ms, and 250–300 ms. The /ɹɑ/ deviants elicited significantly less low gamma neural synchrony over the left hemisphere, as compared to the right. *Significant effects.

Individual participants’ mean low gamma band responses to /ɹɑ/ and /wɑ/ standards and deviants are presented in Supplementary Figure 6. Low gamma activation varied across variables and time windows (Figures 5, 7). The laterality of low gamma activation patterns changed over time, as significantly less gamma band activation was found across left hemisphere electrodes as compared to right hemisphere electrodes from 50 to 100 ms (Left: *M* = –0.028, SEM = 0.023; Right: *M* = 0.011, SEM = 0.016; *F*_(1,14)_ = 5.042, *p* < 0.05, *η*^2^ = 0.265) and from 100 to 150 ms (Left: *M* = –0.042, SEM = 0.028; Right: *M* = 0.012, SEM = 0.021; *F*_(1,14)_ = 6.030, *p* < 0.03, *η*^2^ = 0.301).

The trial type × laterality interaction was significant from 50 to 100 ms (*F*_(1,14)_ = 6.019, *p* < 0.03, *η*^2^ = 0.301) and 100 to 150 ms (*F*_(1,14)_ = 7.589, *p* < 0.02, *η*^2^ = 0.352). The deviants elicited significantly less gamma activation over the left hemisphere than over the right during the 50–100 ms window (*F*_(1,14)_ = 6.152, *p* < 0.03, *η*^2^ = 0.305) and 100–150 ms window (*F*_(1,14)_ = 7.434, *p* < 0.02, *η*^2^ = 0.348), while no laterality difference was observed for the standards. This effect was driven primarily by the /ɹɑ/ deviant responses elicited over the left hemisphere. Specifically, low gamma activation elicited by /ɹɑ/ over the left hemisphere was significantly less than low gamma activation recorded over the right hemisphere during the 100–150 ms window (*F*_(1,14)_ = 5.575, *p* < 0.04, *η*^2^ = 0.285; Figure 7). No laterality differences were noted for the /wɑ/ deviant responses. Moreover, there was a strong trend for the /ɹɑ/ deviants to elicit less low gamma activation than the /wɑ/ deviants over the left hemisphere during both the 50–100 ms (*F*_(1,14)_ = 2.899, *p* < 0.11, *η*^2^ = 0.172) and 100–150 ms windows (*F*_(1,14)_ = 3.063, *p* < 0.10, *η*^2^ = 0.180); no phoneme differences were noted over the right hemisphere.

#### ERSP Summary

The ERSP analyses were exploratory, as previous underspecification work has not addressed this aspect of phonological processing. Thus, the findings are preliminary. Theta band activation was examined to measure syllable-level processing while the low gamma band was examined to measure phoneme-level processing. At the syllable level, /ɹɑ/ elicited greater theta activation than did /wɑ/. At the phoneme level, low gamma activation was significantly lower for /ɹɑ/ vs. /wɑ/ deviants over the left hemisphere, as compared to the right.

## Discussion

This study provides the first neural evidence for [consonantal] underspecification in English-speaking adults. Two phonemes differing in their specification of the [consonantal] feature were contrasted: /ɹ/ and /w/. As /w/ is not specified for [consonantal] while /ɹ/ is, it was hypothesized that asymmetrical speech processing differences would be apparent. Indeed, mean amplitude measurements and cluster permutation analyses both showed that /wɑ/, as an oddball in a sequence of /ɹɑ/, elicited significantly larger MMN responses than did the reciprocal stimulus set—namely, /ɹɑ/ oddballs embedded within frequently occurring instances of /wɑ/. Characterizing the theta and low gamma band neural oscillation patterns provided further evidence for underspecification. The more specified /ɹɑ/ elicited increased activation, or neural synchrony, in the theta bandwidth as compared to /wɑ/. Moreover, the /ɹɑ/ deviants elicited less low gamma activation over the left hemisphere, as compared to the right hemisphere. As neural oscillation patterns have not previously been discussed concerning underspecification, these ERSP analyses identified potentially new indices of phonological underspecification.

### ERP Evidence For [Consonantal] Underspecification

Consistent with previous reports of phonological underspecification (Diesch and Luce, 1997; Eulitz and Lahiri, 2004; Cornell et al., 2011, 2013; Scharinger et al., 2012; Schluter et al., 2016; Cummings et al., 2017), ERP evidence for phonological underspecification was observed. The underspecified /wɑ/ elicited larger neural responses than did more specified /ɹɑ/. Moreover, the cluster permutation analyses identified a significant difference between the /wɑ/ standards and deviants, indicative of a reliable MMN response. No significant difference was observed between the /ɹɑ/ standards and deviants. Thus, the /wɑ/ deviant response (elicited within the /ɹɑ/ standard) appeared to drive the phoneme underspecification differences. These findings were consistent with the underspecification logic of FUL (Eulitz and Lahiri, 2004) which predicts an underspecified phoneme deviant (i.e., /wɑ/) presented within a stream of specified phoneme standards (i.e., /ɹɑ/) would elicit a large mismatch response due to the contrast in [consonantal] feature specification.

While [consonantal] was the obvious feature that differentiated /ɹ/ and /w/, these two phonemes also differ in terms of their place of articulation, with /ɹ/ being characterized as [coronal: +distributed] and /w/ being characterized as [labial] by (Clements and Hume, 1995; Figure 1). Given the previous investigations of [coronal] underspecification (Eulitz and Lahiri, 2004; Cornell et al., 2011, 2013; Scharinger et al., 2012; Cummings et al., 2017), possibly the place of articulation of these phonemes would affect the neural response patterns.

Based on previous FUL work, /ɹ/ could have arguably constituted the underspecified phoneme due to its [coronal] place. However, [coronal] also has the assigned daughter [+distributed] within the Clements and Hume (1995) model^11^. This dependent feature is on a lower level of the feature tree than that of /w/’s [labial]. As features lower on the tree are more specified than those higher up in the tree (Core, 1997), in the Clements and Hume (1995) model, /ɹ/ is more specified in terms of place of articulation [coronal: +distributed], as well as in the manner of articulation [+consonantal]. If the [labial] of /w/ was considered to be underspecified as compared to the [coronal: +distributed] of /ɹ/, this would be contrary to all previous work proposing [coronal] underspecification. As a result, it is hypothesized that the place of articulation was not the target feature contrast of /ɹ/ and /w/. However, the multiple features that are underspecified in /w/ (i.e., [–consonantal] and [labial]), make it unclear as to what exactly might have been the feature that was driving the observed MMN asymmetry.

Additional studies contrasting liquid and glide phonemes are necessary to further test [consonantal] underspecification. Since /ɹ/ and /w/ differ not only in terms of [consonantal] but also in terms of place of articulation, a contrast that only varies [consonantal] is needed. This contrast is possible in /l/ and /j/. Both phonemes are [coronal] in nature, thus their main feature distinction is [consonantal], with /l/ being [+consonantal] ([−vocoid]) and /j/ [–consonantal] ([+vocoid]). Importantly, similar to /ɹ/, prevocalic /l/ often undergoes the phonological process of liquid gliding during typical and atypical phonological development, with /l/ substituted with [j] and/or [w]. For example, young children commonly produce “like” (i.e., /lɑIk/) as [jɑIk]. Thus, both liquid phonemes in American English are commonly observed to undergo liquid gliding during phonological development. These developmental and clinical observations provide additional evidence for the possibility that both American English glides, /w/ and /j/, are underspecified as compared to the American English liquids /ɹ/ and /l/. Replication of the present study with /l/ and /j/ would provide important converging evidence for the underspecification of [consonantal] in glide phonemes.

### ERSP Evidence For [Consonantal] Underspecification

Since EEG neural oscillation patterns drive ERP responses, it was hypothesized that they could be additional indices of phonological underspecification. Exploratory analyses were conducted to examine whether specified and underspecified phonemes elicited distinct patterns of neural activity. Indeed, significant differences in neural oscillation patterns were elicited by /ɹɑ/ and /wɑ/. In the theta band, /ɹɑ/ elicited more spectral power than did /wɑ/. It has been proposed that inherent, resting-state oscillations in the primary auditory cortex undergo phase resetting—particularly in the theta range—in response to speech stimuli (Ghitza, 2011; Giraud and Poeppel, 2012). Thus, the enhanced theta activities between 100 and 300 ms to /ɹɑ/ relative to /wɑ/ likely reflect the impact of specification on this phase resetting process. Within the FUL framework, a more specified phoneme contains more exact phonetic feature information in its phonological representation—which may drive more precise theta phase-locking to the presentation of /ɹɑ/ syllables, yielding a stronger evoked response as compared to an underspecified phoneme that does not contain the same degree of robust featural specification.

As theta activities are proposed to capture syllable-level processing (Ghitza, 2011; Giraud and Poeppel, 2012), a secondary interpretation of the theta band results is acoustic in nature. That is, /ɹɑ/ may have been acoustically more distinct, with a clearer syllable onset boundary, than was /wɑ/. As the sharpness of a syllable’s acoustic edges affects how easily the stimulus can be parsed into chunks (Prendergast et al., 2010; Ding and Simon, 2014; Doelling et al., 2014), /ɹɑ/ was able to elicit greater theta neural synchrony than /wɑ/. To explain further, /ɹ/ is a more preferable syllable onset consonant than /w/ due to the sounds’ sonority differences (Clements, 1990). Specifically, listeners prefer syllables with strong consonant onsets that are clearly differentiated from the vowel nucleus (e.g., the Head Law; Vennemann, 1988). Since /w/ is nearly as sonorous as vowels, it does not provide a clear differentiated onset; thus, syllable-initial /ɹ/ is preferred over /w/ cross-linguistically (Dziubalska-Kołaczyk, 2001). This acoustic interpretation is still consistent with the idea of feature specification, as the [consonant] aspect of /ɹ/ is what arguably makes it a stronger syllable onset than that of /w/. Thus, while /w/ can function as a syllable onset (Bernhardt and Stoel-Gammon, 1994), /ɹɑ/ is a better-formed syllable than /wɑ/ because of its specified [consonant] feature.

While theta band activity has been correlated with syllable-level processing, low gamma band has been correlated with more rapid information sampling, analysis, and decoding (Poeppel, 2003; Ghitza, 2011; Giraud and Poeppel, 2012), likely linked to the binding of different acoustic features needed to derive phonological representations from incoming speech signals. Notably, low gamma band responses have not been consistently observed in auditory paradigms (Luo and Poeppel, 2007; Howard and Poeppel, 2010; Luo et al., 2010), potentially due to the stimuli used (Luo and Poeppel, 2012). The present study provided an ideal situation for eliciting distinguishing gamma responses, as [consonantal] was the contrasting feature between the phonemes.

Our findings revealed less low gamma activation over the left hemisphere as compared to the right hemisphere overall. Specifically, the low gamma activation in response to the /ɹɑ/ deviants was reliably less over the left hemisphere, as compared to the right, whereas the /wɑ/ deviants did not elicit laterality differences. Moreover, /wɑ/ elicited greater low gamma activation over the left hemisphere as compared to /ɹɑ/, while no phoneme differences were observed over the right hemisphere. Thus, /ɹɑ/ appeared to elicit a distinct pattern of activation over the left hemisphere. Interpreting this finding is challenging, given the lack of prior findings. However, a general interpretation could be similar to that of the MMN results. Namely, /wɑ/ elicited greater low gamma activation over the left hemisphere due to its underspecified nature. Future studies will need to continue to test the relationship between underspecification and low gamma activation.

### Alternative Interpretations and Study Limitations

While the data in the present study provide evidence of [consonantal] underspecification, other interpretations are possible. For example, a memory/usage-based account of language (UBA; Pierrehumbert, 2006; Bybee, 2010) addresses how the neighborhood density of phonemes affects processing. That is, the larger a phoneme’s phonological neighborhood, the more difficult it is to identify and differentiate a specific phoneme from others within the neighborhood. Within UBA, the [+consonantal] category contains many more consonants (21: /p b t d k g f v θ ð s z ⎰ ℨ tʃ dʒ m n ŋ l ɹ/) than does the unspecified [–consonantal] category (3: /w j h/). Thus, /ɹ/ has a denser phonological neighborhood than does /w/. When considering MMN responses in the context where /ɹɑ/ is the standard and /wɑ/ is the deviant, UBA would predict that the large phonological neighborhood of /ɹ/ would negatively impact the system’s ability to create a strong feature prediction of [+consonantal]. Without clear feature specification, this situation should result in a no mismatch situation and a small/no MMN being elicited by the /wɑ/ deviant. Conversely, in the context where /wɑ/ is the standard and /ɹɑ/ is the deviant, UBA would predict that the small phonological neighborhood of /w/ would allow the system to establish a strong feature prediction. This should result in a true mismatch situation—and also in a large MMN being elicited by the /ɹɑ/ deviant. However, neither one of these proposed results was observed in the present study. Instead, the exact opposite MMN response patterns were observed. Thus, it does not appear that UBA can account for the present study’s findings.

The frequency occurrence of sounds in the ambient language environment could have unintentionally affected the MMN responses observed in the present study. Specifically, the MMN can reflect the phonotactic probability of phoneme combinations (Bonte et al., 2005; Näätänen et al., 2007). That is, the statistical regularity of sound combinations in a language can modulate the size of the MMN response. For example, nonwords with high phonotactic probability have been found to elicit larger MMN responses than nonwords with low phonotactic probability (Bonte et al., 2005). Bonte et al. (2005) suggested that the frequent co-occurrence of certain phoneme combinations could result in enhanced auditory cortical responses. In the present study, the phonotactic probability of the /ɹɑ/ syllable in English was greater than that of /wɑ/ (Table 1). Thus, following the results of Bonte et al. (2005), the more frequently occurring /ɹɑ/ should have elicited a larger MMN than did /wɑ/, which was not observed. The same general argument could be made for the frequency of occurrence of single phonemes, with /ɹ/ occurring much more frequently in English than /w/ (Table 1). However, again, the high frequency of /ɹ/ did not elicit larger MMN responses than did the less commonly occurring /w/. While the findings of the present study do not appear to be driven by the frequency of occurrence of the phonemes, this will remain a possible interpretation until this prediction is directly tested. Fully-crossed stimulus sets with similar individual phoneme and syllable phonotactic probabilities should be used to elicit responses from high and low frequency phonemes and syllables.

The present study included identity difference waves to control for basic differences in acoustic detail present in the /ɹɑ/ and /wɑ/ stimuli. However, it is still a possibility that the study design and/or stimuli did not test phonological representations, but rather tested the phonetic differences between the stimuli. It has been suggested that a single-standard MMN experiment can only capture the phonetic differences between speech sounds. That is, if the standards are not varied, the established memory trace is based on the consistent phonetic makeup of the standard. It has been argued that a variable-standards MMN experimental design (e.g., /t/ produced with multiple voice onset time allophones) is necessary instead to establish a true phonemic MMN (Phillips et al., 2000; Hestvik and Durvasula, 2016). For example, Hestvik and Durvasula (2016) only observed an underspecification MMN asymmetry using a variable-standards paradigm; symmetrical MMN responses were elicited with a single-standards paradigm.

While the possibility remains that the present study only captured phonetic differences between /ɹɑ/ and /wɑ/, the data suggest that the phonological level of representation was tested. The previous MMN studies accessing phonological representations only used a single deviant within their multiple-standard presentations (Phillips et al., 2000; Hestvik and Durvasula, 2016). Although the present study used a single-standard paradigm, it did incorporate three phoneme deviants. The three deviants were included to maximize the MMN responses. That is, the response to a deviant is reduced not only when it is preceded by itself, but also when it is preceded by other similar stimuli (Sams et al., 1984; Näätänen et al., 2007; Symonds et al., 2017). However, the reduction in MMN amplitude can be reduced if the second of two successive deviants differs from the standards in a different attribute/feature than the first deviant (Nousak et al., 1996; Müller et al., 2005; Näätänen et al., 2007). The two unused deviants in the present study, /bɑ/ and /dɑ/, were chosen in part because they were phonetically distinct from /ɹ/ and /w/. Thus, the presentation of multiple deviants, and the phonetic distinctiveness of the stimuli, could have allowed for phonological categorization to occur. Indeed, unlike Hestvik and Durvasula (2016), asymmetrical MMN responses were found in the present study, indicative of phonological-level processing.

A basic stimulus difference could also explain why a phonological mismatch asymmetry was elicited, rather than the symmetrical phonetic mismatch response predicted by previous studies. That is, previous studies used synthetic speech, while the present study used naturally-produced syllables. The acoustic-phonetic structure of synthetic speech conveys less information (per unit of time) than that of natural speech (Nusbaum and Pisoni, 1985). As a result, synthetic speech is considered to be perceptually impoverished as compared to natural speech because basic acoustic-phonetic cues are obscured, masked, or physically degraded in some way. Natural speech is highly redundant at the level of acoustic-phonetic structure, with many acoustic cues being present in the signal. As limited acoustic information is present in synthetic speech, some phonetic feature distinctions are minimally cued. This means that a single cue presented within a single synthetic stimulus might not be enough to convey a particular level of feature distinction. As a result, multiple different tokens of a synthetic phoneme might need to be presented to fully establish a phonemic category. This hypothesis is supported by the results of the previous studies (Phillips et al., 2000; Hestvik and Durvasula, 2016). Alternatively, the spectral variation and redundancy found in the naturally produced speech tokens of the present study might have been enough to accurately establish phonemic categories.

Thus, the naturally produced standard and deviants in the present study could have allowed for phonological categorization of all the stimuli, much like the variable standard presentation of synthetic speech did in previous studies. That said, it is still a possibility that the memory trace tested in the present study was a detailed acoustic/phonetic representation rather than a phonemic representation. Future studies that systematically vary the phonetic allophonic productions and phonemic categories of both standards and deviants are needed to address how best to access phonological representations. Additional studies contrasting synthetic and naturally-produced speech will also provide information regarding how specified and unspecified features are stored and accessed.

As discussed previously concerning theta band activities, possibly the acoustic differences of /ɹɑ/ and /wɑ/ alone were responsible for the observed MMN response asymmetry. That is, the intrinsic physical differences between stimuli could elicit different MMN response patterns (Näätänen et al., 2007). For example, the larger sonority difference between /ɹ/ and /ɑ/ made it an acoustically more distinctive syllable than that of /wɑ/^12^. In other words, the /w/ is perceptually more similar to /ɑ/ than is /ɹ/. Thus, if acoustic distinctiveness and clarity were the underlying mechanisms driving the MMN responses, hearing the deviant /ɹɑ/ within a stream of the /wɑ/ standards should have elicited a larger MMN response than hearing the deviant /wɑ/ in the stream of /ɹɑ/ standards. Yet, the opposite MMN response pattern was observed. The less acoustically distinct /wɑ/ deviant elicited a larger MMN than did the acoustically preferable /ɹɑ/. Moreover, the MMN response elicited by both syllables was larger over the left hemisphere, as compared to the right, which is indicative of feature-level processing; acoustical change detection would have been indicated by similar bilateral MMN responses (Näätänen et al., 1997).

While underspecification is presumed to be a language universal phenomenon, possibly the specification of features can vary across languages. For example, voiced stops are underspecified in English, while voiceless stops are underspecified in Japanese (Hestvik and Durvasula, 2016; Hestvik et al., 2020). In terms of /ɹ/, Natvig (2020) proposed that liquids, and rhotics in particular, are underspecified consonantal sonorants due to the multiple variations of “r-sounds” that occur in languages such as German, Arabic, Hawaiian, New Zealand Maori, Malayalam, and Norwegian. While it is beyond the scope of this study to address whether /ɹ/ is specified or not in languages other than English, cross-linguistic differences in [consonantal] underspecification are possible.

The decision to use /ɹ/ and /w/ here was driven by the need to better understand the clinical observation of particular speech error patterns observed during phonological development. Specifically, young typically developing children, as well as older children with speech sound disorders, often have difficulty producing /ɹ/ with adequate palatal and pharyngeal constriction, resulting in an incorrect [w] production. Thus, it was hypothesized that constriction [i.e., (consonantal)] is the primary distinguishing feature of /ɹ/ and /w/, at least in American English. Clements and Hume (1995) feature geometry theory was used to address the underlying differences in the phonological representations of /ɹ/ and /w/. Alternative explanations, including usage-based phonology, phonotactic probability, and sonority/acoustics/phonetics were explored. However, none of the predictions made by these approaches fit with the data. Moreover, while it was possible that [labial] underspecification of /w/ elicited the observed results, that explanation would not be consistent with the many previous studies showing [coronal] to be the underspecified place of articulation. As a result, the presence or absence of [consonantal] in the phonological representations of /ɹ/ and /w/, respectively, is the current best explanation of the results. Future work can either further confirm and extend our proposal, or correct it as needed.

FUL’s underspecification predictions, tested within an oddball paradigm, provide a clear framework within which to examine feature encoding and the specification of phonological representations. By contrasting single phonemes, different patterns of neural responses can be associated with distinctive features. The identification of individual features’ neural patterns is a necessary first step in understanding how speech perception and processing lead to language comprehension and production. However, as pointed out by a reviewer, the use of individual phonemes and/or syllables in the oddball paradigm does not capture the complexity of parsing phonemes (and features) and their subsequent mapping onto lexical items in single words or continuous speech (Gwilliams et al., 2018, 2020; Dikker et al., 2020). To further understand how phonological underspecification improves the efficiency of speech processing, studies involving naturalistic language tasks are an important next step.

### Underlying Neural Mechanisms for Underspecification

From its theoretical inception, underspecification has been proposed as a mechanism to improve the efficiency^13^ of speech processing (Chomsky and Halle, 1968; Kiparsky, 1985; Archangeli, 1988; Mohanan, 1991; Clements and Hume, 1995; Steriade, 1995; Eulitz and Lahiri, 2004). That is, an underspecified feature is the default in a phonological representation. It is efficient to assume a feature is underspecified unless evidence is presented to the contrary. The predictability of that default status allows for ease of phonological processing.

The hallmark neural index of underspecification in electrophysiological studies has been a larger MMN to underspecified phonemes, as compared to specified ones. However, few proposals have been made to address the underlying neural mechanisms of this underspecification response. The size of the MMN has been associated with ease of discrimination (Tiitinen et al., 1994; Näätänen et al., 1997). The large underspecification MMN response would thus suggest that it is easier to discriminate an underspecified feature in a phoneme within a stream of specified phonemes, as compared to contrasting a specified feature within a stream of underspecified phonemes. But what does this large MMN response characterize at a neural level?

From a neurophysiological standpoint, one possibility is that the size of the MMN reflects the tuning characteristics of the responding neural populations. That is, the specification of a feature could lead to the recruitment of specialized neural populations that are tuned to only respond to that feature. Conversely, if a phoneme is not specified for a feature, other less-specialized populations of neurons could be recruited to respond. These less-specialized neurons could be weakly tuned for phonetic-acoustic content. By having weaker encoding, these neurons might be more flexible in their perceptual responses and would likely respond to more types of features at the same time. As a result, the responses elicited by the less-specified neurons could be larger than those of the specifically tuned neurons because they are coded to respond to more types of acoustic-phonetic information. Besides, since the less-specified neurons might be activated more frequently due to their lack of feature specification, their responses could be more highly tuned/practiced, which could also result in larger responses.

In regards to the present study, perhaps the underspecified [–consonantal] feature in /wɑ/ could activate the weakly-coded neurons that were tuned to respond to a variety of phonetic-acoustic content. This broad phonetic-acoustic tuning could elicit a large neural response due to the many different cues that might be summed together in the response. Alternatively, the specified feature in /ɹɑ/ could access neuronal populations that were explicitly coded for a single feature, [+consonantal]. Thus, the neurons would respond, but only to that specific feature and ignore all other features. This could result in a small neural response.

Neuroimaging studies have provided some evidence in support of this proposal. For example, very small populations of neurons (characterized by single electrodes or voxels) have been found to encode and respond to linguistically meaningful information, such as formant frequencies (e.g., low-back vowels), phonetic features (e.g., obstruent, plosive, voicing), and/or entire phonemes (Mesgarani et al., 2014; Arsenault and Buchsbaum, 2015; de Heer et al., 2017; Gwilliams et al., 2018; Yi et al., 2019). Also, phonemes and features elicited activation across multiple electrodes and voxels, suggesting that responses were not constrained to a single neural population. Thus, there is evidence for highly tuned neural populations to respond to one, or many, features, while also working in conjunction with other neural populations.

To our knowledge, previous studies of underspecification have not directly discussed the neural implications of underspecification, and rightfully so, given the limited spatial resolution of scalp-level EEG recordings (Luck, 2014). The present study proposes some possible neural-level interpretations of its results. Future collaborative work with researchers using spatially sensitive neuroimaging techniques will be necessary to further define the underlying neural mechanisms of underspecification.

### Summary and Conclusions

The less specified /wɑ/ elicited a large MMN, whereas a much smaller MMN was elicited by the more specified /ɹɑ/. This outcome reveals that the [consonantal] feature follows the underspecification predictions of FUL previously tested with the place of articulation and voicing features. Thus, this study provides new evidence for the language universality of underspecification by addressing a different phoneme feature. Moreover, left hemisphere low gamma activation characterized distinct phoneme-specific feature processing patterns for /ɹ/ and /w/, revealing a potentially novel index of underspecification. Examining theta and/or low gamma bandwidths in future studies could provide further support for the claims of underspecification.

## Data Availability Statement

The raw data supporting the conclusions of this article will be made available by the authors, without undue reservation.

## Ethics Statement

The studies involving human participants were reviewed and approved by Idaho State University Human Subjects Committee and the University of North Dakota Institutional Review Board. The patients/participants provided their written informed consent to participate in this study.

## Author Contributions

AC created the stimuli, tested participants, prepared and analyzed the data, and helped write the manuscript. DO and YW analyzed data and helped write the manuscript. All authors contributed to the article and approved the submitted version.

## Conflict of Interest

The authors declare that the research was conducted in the absence of any commercial or financial relationships that could be construed as a potential conflict of interest.

## Footnotes

^1^The /ɹ/ is generally considered to be more complex and specified than /w/ (Greenberg, 1975) as it is acquired later in development (McLeod and Crowe, 2018) and occurs in fewer world languages than /w/ (Maddieson, 1984).

^2^There is some debate as to the organization of the tree, but all theories agree upon a root node, and separate class nodes.

^3^Phonotactic probability refers to the frequency with which a phonological segment, such as /ɹ/, and a sequence of phonological segments, such as /ɹɑ/, occur in a given position in a word (Jusczyk et al., 1994).

^4^https://calculator.ku.edu/phonotactic/about

^5^http://www.sccn.ucsd.edu/eeglab

^6^These electrodes encompass four anterior-posterior levels (Frontal, Frontal-Central, Central, Central-Parietal) and three left-right laterality levels [Left (1), Midline (z), Right (2)].

^7^https://openwetware.org/wiki/Mass_UnivariateERPToolbox.

^8^These electrodes encompassed two laterality levels (Left: F3, F1, FC3, FC1, C3, C1, CP3, CP1; Right: F4, F2, FC4, FC2, C4, C2, CP4, CP2), four anterior-posterior levels (Frontal, Frontal-Central, Central, Central-Parietal) and two electrode laterality levels (Far laterality: F3/F4, FC3/FC4, C3/C4, CP3/CP4; Close laterality: F1/F2, FC1/FC2, C1/C2, CP1/CP2).

^9^Note that while only the mismatch responses elicited by /wɑ/ were found to be significant *via* the cluster permutation analyses, mismatch responses were also present in the /ɹɑ/ difference wave (Figure 2).

^10^Note that while only the mismatch responses elicited by /wɑ/ were found to be significant *via* the cluster permutation analyses, mismatch responses were also present in the /ɹɑ/ difference wave (Figure 2).

^11^The LN is a negativity that follows the MMN and typically peaks between 300 and 500 ms at fronto-central electrode sites.

^12^It should be noted that this is a different feature assignment than what would be found in FUL (Lahiri & Reetz, 2002), as there are no articulator dependents in FUL; this is what allows for [coronal] to be underspecified. The dependents in the Clements and Hume (1995) make it difficult, or impossible, for [coronal] to be underspecified.

^13^It is important to note that the acoustic aspects of cannot be fully differentiated from language experience and phonotactics as honeme combinations that are perceptually more distinct tend to occur more often in and across languages (Bonte et al., 2005).
