# Supplementary material for: Phonological Underspecification: An Explanation for How a Rake Can Become Awake
Source: Front Hum Neurosci. 2021 Feb 17;15:585817. doi: 10.3389/fnhum.2021.585817 (PMC7925882; doi:10.3389/fnhum.2021.585817)

Supplementary Figure 1. Event-related spectral perturbation activation patterns (in dB) elicited by the /wɑ/ and /ɹɑ/ standard and deviant (combined) stimuli in 16 electrodes for theta (4-7 Hz), alpha (8-12 Hz), and low gamma (25-35 Hz) bandwidths. Time is on the x-axis and frequency is on the y-axis.

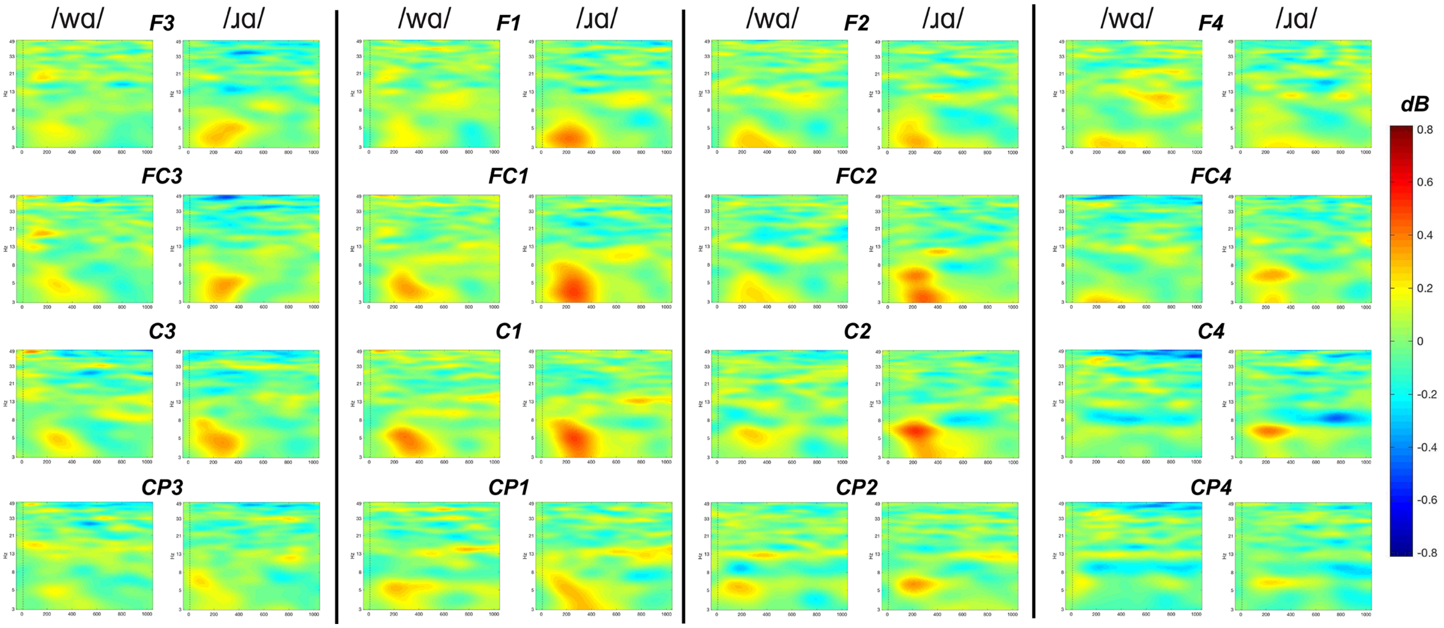

Supplement: Supplementary file 1 [file Data_Sheet_1.PDF]
