# Supplementary material for: Phonological Underspecification: An Explanation for How a Rake Can Become Awake
Source: Front Hum Neurosci. 2021 Feb 17;15:585817. doi: 10.3389/fnhum.2021.585817 (PMC7925882; doi:10.3389/fnhum.2021.585817)

**Supplementary Figure 2.** Scatterplots highlighting the variation in individual participants' mean ERP amplitudes in three 50 ms time windows: 100-150 ms, 150-200 ms, and 200-250 ms. Row A displays /wɑ/ and /ɹɑ/ identity difference waves, Row B displays /wɑ/ standard and deviant ERPs, and Row C displays /ɹɑ/ standard and deviant ERPs. All responses are averaged across the 12 electrodes included in the mean amplitude analyses. The relationship between the /wɑ/ and /ɹɑ/ MMN responses decreased over time while the relationship between the standards and deviants of each stimulus increased with time.

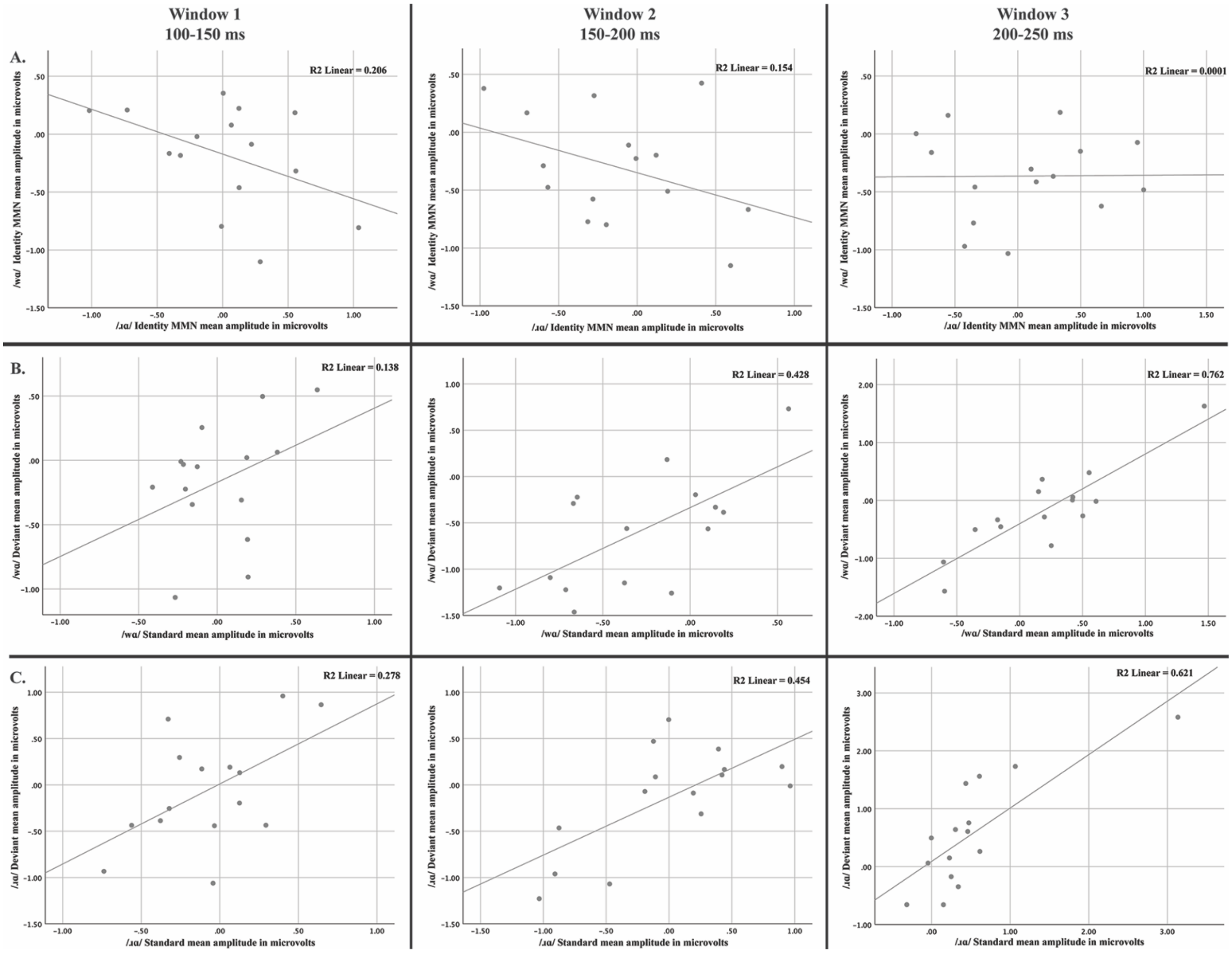

Supplement: Supplementary file 2 [file Data_Sheet_2.PDF]
