# Supplementary material for: Phonological Underspecification: An Explanation for How a Rake Can Become Awake
Source: Front Hum Neurosci. 2021 Feb 17;15:585817. doi: 10.3389/fnhum.2021.585817 (PMC7925882; doi:10.3389/fnhum.2021.585817)

Supplementary Figure 3. Identity difference waveforms elicited by /wɑ/ and /ɹɑ/ across the 12 electrodes included in all analyses. The overall magnitude of the MMN was significantly larger over the left hemisphere, as compared to the right, during the 150-200 ms time window.

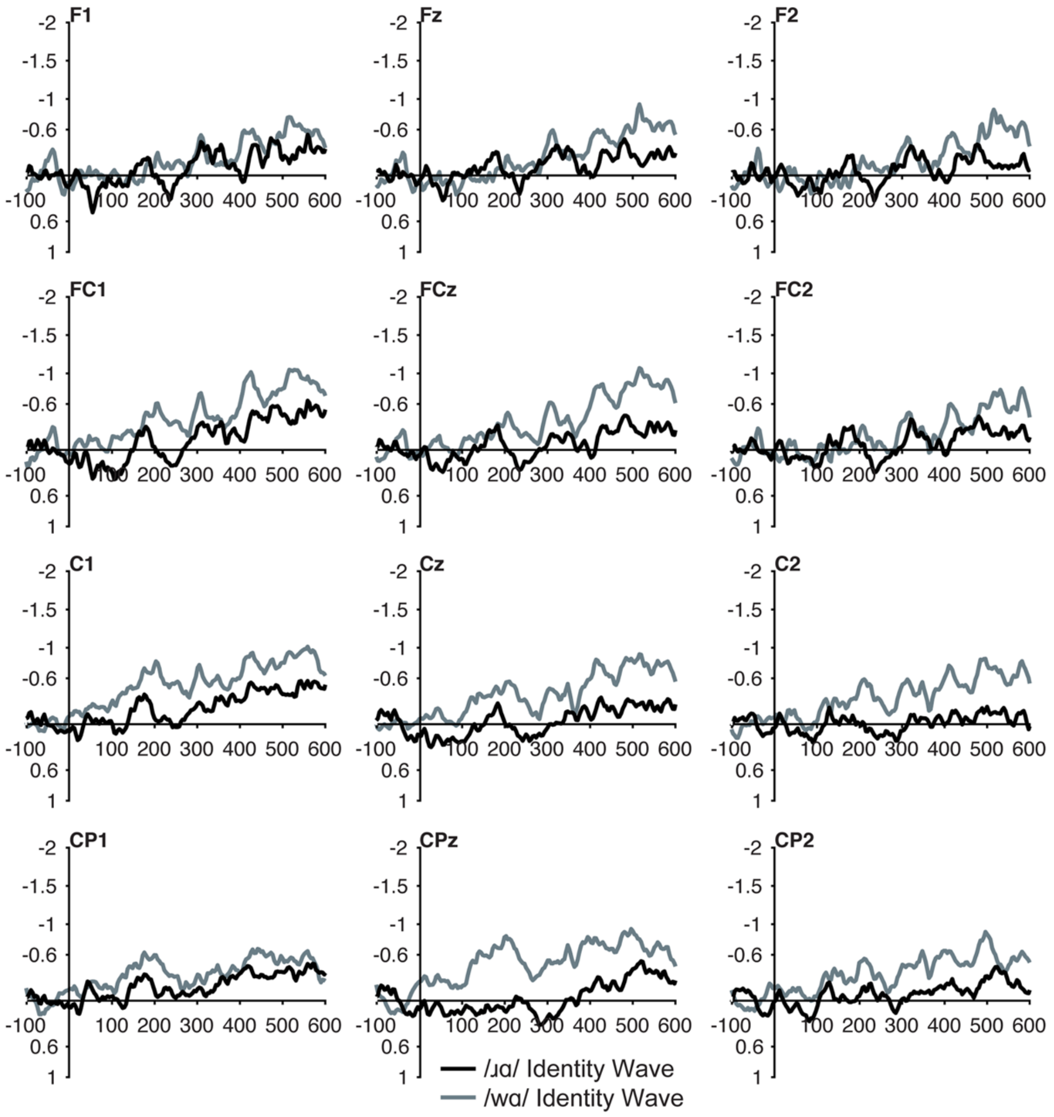

Supplement: Supplementary file 3 [file Data_Sheet_3.PDF]
