# Supplementary material for: Phonological Underspecification: An Explanation for How a Rake Can Become Awake
Source: Front Hum Neurosci. 2021 Feb 17;15:585817. doi: 10.3389/fnhum.2021.585817 (PMC7925882; doi:10.3389/fnhum.2021.585817)

Supplementary Figure 5. Scatterplots highlighting the variation in individual participants' theta (4-7 Hz) bandwidth responses in the 100-300 ms time window. The /wɑ/ standard and deviant responses at the top of the figure and /ɹɑ/ standard and deviant responses on the bottom of the figure. All responses are averaged across the 16 electrodes included in the theta bandwidth ERSP analyses. As there were no significant differences in trial type, the correlational relationship between the standards and deviants for each stimulus was small. Evidence for the greater theta activation to /ɹɑ/ can be seen in the amplitude differences of the individual participants.

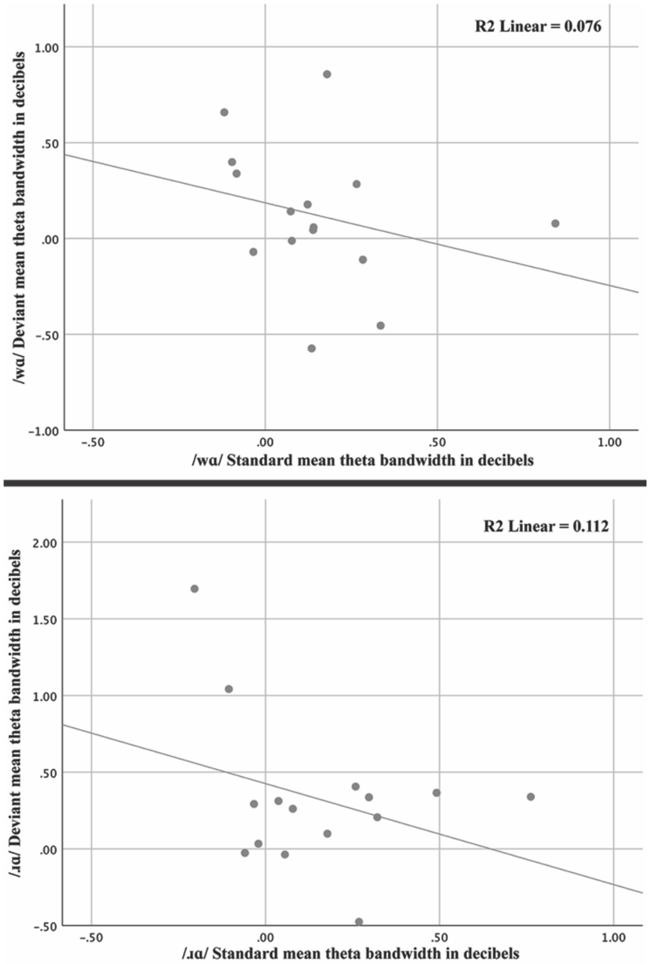

Supplement: Supplementary file 5 [file Data_Sheet_5.PDF]
