# Supplementary material for: Phonological Underspecification: An Explanation for How a Rake Can Become Awake
Source: Front Hum Neurosci. 2021 Feb 17;15:585817. doi: 10.3389/fnhum.2021.585817 (PMC7925882; doi:10.3389/fnhum.2021.585817)

Supplementary Figure 6. Scatterplots highlighting the variation in individual participants' low gamma (25-35 Hz) bandwidth responses in five 50 ms time windows: 50-100 ms, 100-150 ms, 150-200 ms, 200-250 ms, and 250-300 ms. The /wɑ/ standard and deviant responses at the top of the figure and /ɹɑ/ standard and deviant responses on the bottom of the figure. All responses are averaged across the 16 electrodes included in the low gamma bandwidth ERSP analyses. The correlational relationship between the low gamma responses elicited by the standards and deviants for each stimulus decreased over time, with the highest correlations occurring in the earliest time window. Moreover, the correlation between the /wɑ/ standard and deviant was higher than that of the /ɹɑ/ standard and deviant. Evidence for the greater low gamma activation to /wɑ/ can be seen in the amplitude differences of the individual participants across all time windows.

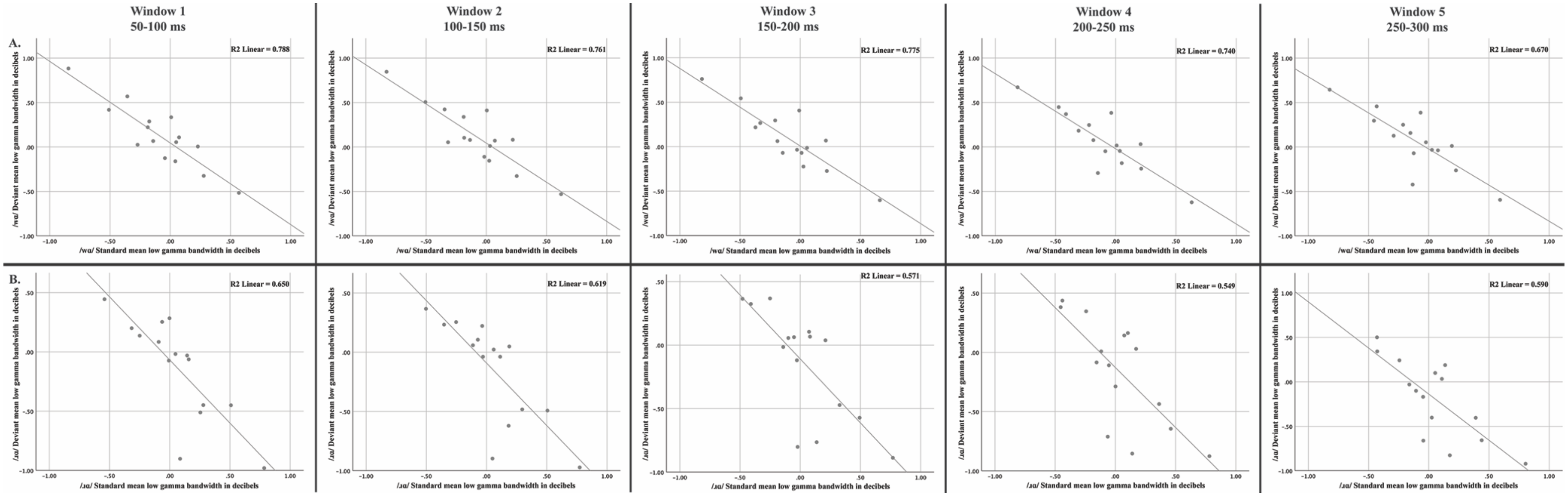

Supplement: Supplementary file 6 [file Data_Sheet_6.PDF]
